# Urine neutrophil gelatinase-associated lipocalin: a diagnostic and prognostic marker for acute kidney injury (AKI) in hospitalized cirrhotic patients with AKI-prone conditions

**DOI:** 10.1186/s12876-015-0372-5

**Published:** 2015-10-16

**Authors:** Sombat Treeprasertsuk, Amornpun Wongkarnjana, Veeravich Jaruvongvanich, Sasipim Sallapant, Khajohn Tiranathanagul, Piyawat Komolmit, Pisit Tangkijvanich

**Affiliations:** 1 Division of Gastroenterology, Department of Medicine, Faculty of Medicine, and King Chulalongkorn Memorial Hospital, Chulalongkorn University, Rama4 Road, Pathumwan District, Bangkok 10330 Thailand; 2 Division of Nephrology, Department of Medicine, Faculty of Medicine, and King Chulalongkorn Memorial Hospital, Chulalongkorn University, Bangkok, 10330 Thailand; 3 Department of Biochemistry, Faculty of Medicine, and King Chulalongkorn Memorial Hospital, Chulalongkorn University, Bangkok, 10330 Thailand

**Keywords:** Urine neutrophil gelatinase-associated lipocalin, Cirrhosis, Acute kidney injury, Diagnostic marker, Prognostic marker

## Abstract

**Background:**

Acute kidney injury (AKI) is known to increase mortality in hospitalized cirrhotic patients; therefore early identification is utmost significance. There are only a few studies evaluating the cut-off level of urine neutrophil gelatinase-associated lipocalin (uNGAL) for diagnosing AKI and its prognostic value in cirrhotic patients. We aimed to determine the accuracy of uNGAL as a biomarker for early identification of AKI and to determine the cut-off level of uNGAL for diagnosing AKI in hospitalized cirrhotic patients; and (2) to explore the association of 30-day liver-related mortality with uNGAL level.

**Methods and Material:**

We prospectively enrolled cirrhotic patients admitted at the King Chulalongkorn Memorial Hospital during May 1, 2011 to Dec 31, 2013. UNGAL levels were measured within 24 h after admission. Clinical and laboratory data were obtained. Patients were followed up to 30 days.

**Results:**

Of 137 cirrhotic hospitalized patients, 121 cirrhotic patients (88.3 %) with AKI-prone conditions were included with mean age of 57.3 ± 14.7 years. Thirty-five patients (29 %) developed AKI within 72 h of admission. The causes of AKI were prerenal azotemia (68.6 %), acute tubular necrosis (25.7 %), hepatorenal syndrome (5.7 %), respectively. The mean uNGAL level was significantly higher in the patients who developed AKI compared with those who did not (290.6 ± 356.3 vs. 54.4 ± 73.7 ng/mL; *P* = 0.0001). The AUC of uNGAL for diagnosing AKI was 0.83 (95 % [CI]: 0.76–0.91) with the optimal cut-off level of 56 ng/mL, providing 77.1 % sensitivity and 73.3 % specificity. Fourteen percent of subjects died during the 30-day follow-up period. The mean uNGAL levels were significantly higher in the mortality group. The AUC of uNGAL in predicting mortality was 0.75 (95 % [CI]: 0.66–0.85), with a best cut-off level of 72 ng/mL providing 70.6 % sensitivity and 69.2 % specificity. However, in multivariate logistic regression analysis, uNGAL is not an independent factor for 30-day liver-related mortality prediction.

**Conclusions:**

uNGAL is a valid marker for the early detection of AKI in hospitalized cirrhotic patients with AKI-prone conditions; however, its level could not independently predict 30-day liver-related mortality.

## Background

Patients with cirrhosis frequently require hospitalization. Acute kidney injury (AKI) is a common complication occurring up to 20 % in hospitalized cirrhotic individuals [1] and associated with four-fold increased risk of mortality [2]. In clinical practice, serum creatinine and urine output are used as indicators of renal dysfunction, despite their known limitations, especially in advanced cirrhotic group [3–5]. Serum creatinine does not only reflect the renal dysfunction but also the degree of liver dysfunction and malnutrition [4]. Moreover, the rising of serum creatinine lags behind the onset of AKI at least 24 h, which limit its sensitivity and previous study found that the severity of renal injury associated with mortality in hospitalized cirrhotic patients so prompt diagnose and provide early treatment are utmost significance [6, 7].

Urine neutrophil gelatinase-associated lipocalin (uNGAL) has emerged as a potential diagnostic biomarker for AKI. It is a 25-kD polypeptide that is upregulated and secreted in an early stage of AKI [8]. In experimental and clinical studies, uNGAL has been extensively investigated in range of clinical settings [9–13]. Recent studies have focused on the utility of this marker in cirrhotic patients. However, most studies investigated in patients who had renal injury before admission demonstrating that uNGAL could differentiate between subtypes of renal injuries including acute tubular necrosis (ATN), hepatorenal syndrome (HRS), and prerenal azotemia (PRA) [14–16]. Only a few studies investigated this marker for AKI that developed during admission as follows: Slack, A. J. et al. demonstrated that uNGAL was significantly higher in AKI versus non-AKI but their cohort recruited only small number of samples [17]. The other study (Belcher, J. M et al.) used this marker to differentiate ATN versus others (HRA, PRA) [18]. However, there was no study investigated the cut-off level of this marker for diagnosing new-onset AKI during admission.

In term of prognostic value of uNGAL, only three studies so far investigated about its mortality prediction and demonstrated mix of results. Verna, E. C. et al. showed that uNGAL was an independent in-hospital mortality predictor in patients who had renal injury prior to admission [15]. Barreto, R. et al. showed that uNGAL was an independent 3-month mortality predictor in AKI with bacterial infection. In contrast, Gungor, G. et al. demonstrated that uNGAL could not predict 90-day mortality in cirrhotic patients with HRS [19]. Further study is needed to verify this association in hospitalized patients with new-onset AKI and focused on liver-related mortality.

Therefore, we performed a cohort study of hospitalized adult cirrhotic patients with AKI-prone conditions. We aimed: (1) to determine the accuracy of uNGAL as a biomarker for the early identification of AKI and to determine the cut-off level of uNGAL for diagnosing AKI in adult hospitalized cirrhotic patients; and (2) to explore the association of 30-day liver-related mortality with uNGAL level.

## Methods

### Study design

This was a prospective study of adult cirrhotic patients who admitted with AKI-prone conditions. All patients had normal baseline serum creatinine within 3 months prior to admission and were enrolled during May 1, 2011 to Dec 31, 2013 at tertiary-care, King Chulalongkorn Memorial hospital. One hundred and thirty-seven patients with cirrhosis, aged more than 18 years, were admitted during the study period. Exclusion criteria were chronic kidney disease, or previous liver or kidney transplantation. The diagnosis of cirrhosis was based on a combination of clinical, biochemical and imaging assessments (ultrasound/computed tomography/magnetic resonance imaging) or liver biopsy.

Clinical data included demographics, cause of cirrhosis, Child-Pugh score, MELD score, use of vasoactive drug, blood transfusion, and length of hospital stay, together with laboratory data included liver function test, complete blood count, coagulogram, and blood culture were collected within 72 h after admission. Patients had urine collection upon enrollment (within 24 h after admission) and at the subsequent 24 h for uNGAL measurements. Blood samples were drawn for serum creatinine upon enrollment and at 24, 48 and 72 h. Patients were prospectively followed up for mortality assessments to 30 days. The mortality causes were liver-related conditions including liver-related infection, HRS, gastrointestinal bleeding, hepatic encephalopathy, liver failure, and hepatocellular carcinoma.

This study was approved by the institutional review board (IRB. number 357/55) of the Faculty of Medicine, Chulalongkorn University, Bangkok, Thailand, and all participants provided permission for their medical information to be used for research purposes. Written informed consent was obtained from all participants.

### Operational definitions of AKI-prone conditions

1. Volume depletion, e.g., gastrointestinal (GI) bleeding, GI fluid loss, over-diuresis, excessive fluid loss in patients with ascites treated with diuretics, large-volume paracentesis [1, 20–22].
2. Bacterial infections, such as spontaneous bacterial peritonitis (SBP), pneumonia, urinary tract infection (UTI), skin and soft tissue infections, hepatobiliary infection, spontaneous bacteremia as defined by standard diagnostic criteria for each specific infection.
3. Recent exposure to nephrotoxic agents, e.g., NSAIDs, contrast agents.
4. Acute decompensated cirrhosis, e.g., hepatic encephalopathy, variceal bleeding, SBP and HRS.

### Definitions of kidney disease

AKI was defined according to the Acute Kidney Injury Network (AKIN) criteria as an increase in serum creatinine ≥ 0.3 mg/dL or 50 % from baseline within 48 h [3]. Renal ultrasound was performed to exclude structural urinary tract obstruction. PRA was diagnosed in patients who had history of volume depletion together with urine sodium less than 20 mEq/L and a FeNa <1 %, and return of serum creatinine to baseline after volume replacement within several days. HRS was defined by the current standard definition for cirrhosis [23]. ATN was diagnosed in patients who had volume depletion or nephrotoxic agent exposure together with urine sodium more than 40 mEq/L and FeNa >2 % [24].

### Measurement of uNGAL

Urine samples for uNGAL testing were immediately centrifuged at 1500 rpm for 10 min, and the supernatant was stored at −70 °C for batched analysis. UNGAL was measured by chemiluminescent microparticle assay using an ARCHITECT platform (Abbott Diagnostics Inc., Abbott Park, IL, expressed as ng/mL).

### Statistical analysis

Categorical data were reported as counts and percentages and compared using Fisher’s exact test. Continuous variables were expressed as the mean and standard deviation (SD). Comparisons between groups were performed by the independent samples *t* test for the values with normal distributions and by the Mann–Whitney (Wilcoxon rank) test for the continuous variables without normal distributions. Normal distribution was determined using the Kolmogorov–Smirnov test. The chi-square test, Fisher’s exact test, and one-way repeated measures analysis of variance (ANOVA) were used as appropriate. All P-values were 2-sided, and *P* < 0.05 was considered significant. The area under the curve (AUC) for each receiver operating characteristic curve was used to quantify the capacity of uNGAL to diagnose AKI and predict the mortality. Receiver operating characteristic (ROC) curves was performed to determine the optimal cut-off values, sensitivity and specificity. To find the optimal threshold point from ROC curve, we used the Youden index criterion. These cutoffs were used in multivariate logistic regression modeling for mortality prediction. Univariate and multivariate logistic regression models were used to evaluate the relationship between uNGAL and the mortality. SPSS for Mac (version 19.0; SPSS Inc., Chicago, IL, USA) was used for statistical analysis.

## Results

### Baseline characteristics of patients

**Fig. 1:**
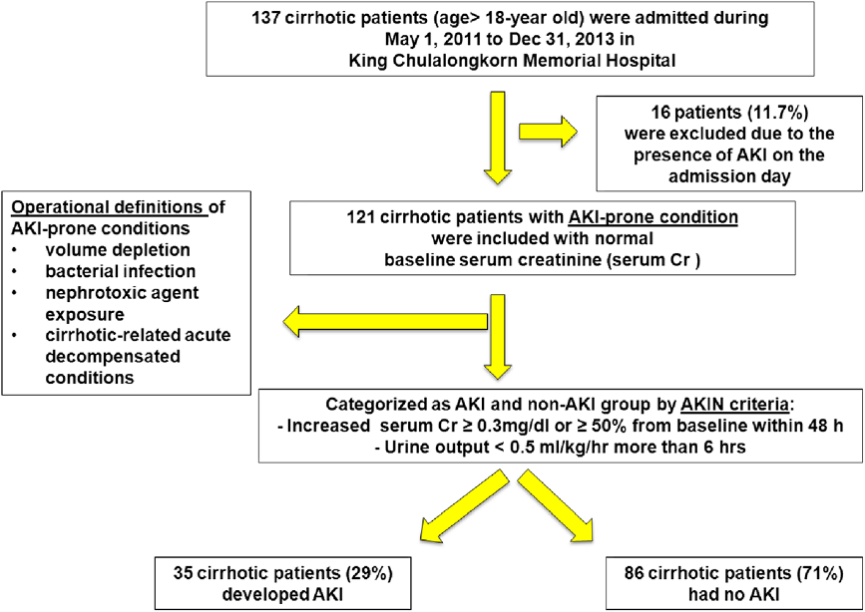
The diagram of inclusion criteria of cirrhotic patients with AKI–prone condition

Of 137 cirrhotic patients who were admitted during the study period, 121 cirrhotic patients (88.3 %) with AKI-prone conditions were included (Fig. 1) and their baseline clinical and laboratory data were detailed in Table 1. The mean age of the patients was 57.3 ± 14.7 years, and 62 % were male. Underlying etiologies of liver injury were chronic hepatitis B/C (52.1 %), alcoholic cirrhosis (26.4 %), cryptogenic cirrhosis (11.6 %), NASH (5.8 %), and autoimmune hepatitis (4.1 %), respectively. The MELD score was 14.9 ± 5.7. Almost 80 % of patients were Child-Pugh B and C. Baseline serum creatinine was 0.9 ± 0.3 mg/dL. Thirty-five cirrhotic patients (29 %) developed AKI within 72 h after admission. In comparison of AKI versus non-AKI subgroup, there was no significant difference in baseline clinical data including Child-Pugh score and MELD score except for higher proportion of ascitic patients was observed in AKI group (71.4 % versus 50.0 %, *P* = 0.03). In term of laboratory data, only ALP (212.9 ± 134.2 U/L versus 142.9 ± 88.7 U/L, *P* = 0.001) and platelet (170.3 ± 197.4/10^3^uL versus 111.3 ± 63.8/10^3^uL) were significantly higher in AKI compared with non-AKI group. Other markers were not significantly different between groups. Of 121 patients with AKI-prone conditions, 64 (53 %) had bacterial infections which were SBP in 16, bacteremia in 14, UTI in 11, hepatobiliary infection in 10, skin and soft tissue infection in 7, respiratory tract infection in 6 cases, respectively (Table 2). The remaining of 57 cirrhotic patients with AKI-prone conditions (47 %) were hospitalized due to GI bleeding (*n* = 32; 26.4 %), acute decompensation from hepatic encephalopathy (*n* = 15; 12.4 %), hypovolemia from diuretic overdose (*n* = 6; 5 %) and hypovolemia post abdominal paracentesis (*n* = 4; 3.3 %).

**Table 1:** Patient characteristics at baseline categorized by the presence or absence of AKI

Variables	Total	No AKI	AKI	*P*
Mean ± SD	(*n* = 121)	(*n* = 86)	(*n* = 35)	
Age (years)	57.3 ± 14.7	56.4 ± 14.7	59.6 ± 14.8	0.27
% Male	75 (62 %)	52 (60.5 %)	23 (65.7 %)	0.59
Cause of cirrhosis				0.74
-Chronic hepatitis B/C	63 (52.1 %)	44 (51.2 %)	19 (54.3 %)	
-Alcohol	32 (26.4 %)	25 (29.1 %)	7 (20.0 %)	
-NASH	7 (5.8 %)	4 (4.7 %)	3 (8.6 %)	
-Cryptogenic	14 (11.6 %)	9 (10.5 %)	5 (14.3 %)	
-Autoimmune	5 (4.1 %)	4 (4.7 %)	1 (2.9 %)	
Child-Pugh				0.20
A	25 (20.7 %)	21 (24.4 %)	4 (11.4 %)	
B	55 (45.5 %)	39 (45.3 %)	16 (45.7 %)	
C	41 (33.9 %)	26 (30.2 %)	15 (42.9 %)	
Ascites	68 (56.2 %)	43 (50.0 %)	25 (71.4 %)	0.03
MELD score	14.9 ± 5.7	14.3 ± 5.9	16.4 ± 5.1	0.08
Serum creatinine	0.88 ± 0.29	0.83 ± 0.29	0.96 ± 0.28	0.05
TB (mg/dl)	4.7 ± 5.1	4.5 ± 4.4	5.2 ± 6.6	0.49
DB (mg/dl)	3.4 ± 4.3	3.2 ± 3.5	4.1 ± 5.8	0.28
AST (U/L)	139.7 ± 250.3	129.9 ± 259.8	163.7 ± 22	0.50
- Median (IQR)	73 (43.5–127)	69.5 (44–119)	82 (35–185)	
ALT (U/L)	78.4 ± 174.5	72.6 ± 168.2	92.9 ± 190.38	0.56
- Median (IQR)	34 (24–53)	33.5 (24–52)	(25–59)	
ALP (U/L)	163 ± 108	143 ± 89	213 ± 134	0.001
INR	1.51 ± 0.77	1.51 ± 0.87	1.53 ± 0.45	0.93
Albumin (g/dl)	2.85 ± 0.86	2.9 ± 0.9	2.7 ± 0.6	0.23
Hemoglobin (g/dl)	10.7 ± 2.4	10.7 ± 2.2	10.6 ± 2.9	0.87
WBC count (10^3^/uL)	9.86 ± 6.2	9.6 ± 6.1	10.4 ± 6.6	0.53
Platelet (/10^3^uL)	128 ± 121	111 ± 64	170 ± 197	0.014

**Table 2:** Indications of hospitalizations of 121 cirrhotic patients and the cause of bacterial infections in 64 cirrhotic patients

Variables	Total	No AKI	AKI
Number (%)	(*n* = 121)	(*n* = 86)	(*n* = 35)
1. Bacterial infections	64 (53)	41 (34)	23 (19)
- SBP^a^	16 (13.2)	10 (11.6)	6 (17.1)
- Bacteremia	14 (11.6)	7 (8.1)	7 (20)
- Urinary tract infection	11 (9.1)	9 (10.5)	2 (5.7)
- Hepatobiliary infection	10 (8.3)	7 (8.1)	3 (8.6)
- Skin and soft tissue infection	7 (5.8)	6 (7)	1 (2.9)
- Respiratory tract infection	6 (5)	2 (2.3)	4 (11.4)
2. No bacterial infections	57 (47)	45 (37.1)	12 (9.9)
- GI bleeding	32 (26.4)	28 (23.1)	4 (0.03)
- Hepatic encephalopathy	15 (12.4)	7 (0.06)	8 (0.07)
- Diuretic overdose	6 (5)	6 (0.05)	0
- Post abdominal paracentesis	4 (3.3)	4 (0.03)	0

SBP*:Spontaneous bacterial peritonitis

### uNGAL and the diagnostic value

Our study measured uNGAL twice, first within 24 h after admission (median uNGAL = 41.2 ng/mL) then at the 24-h interval (median uNGAL = 48.4 ng/mL). The correlation of both values was excellent (*r* = 0.89, *P* < 0.001). Therefore; we used only the first measurement level to analyze its early diagnostic utility.

**Fig. 2:**
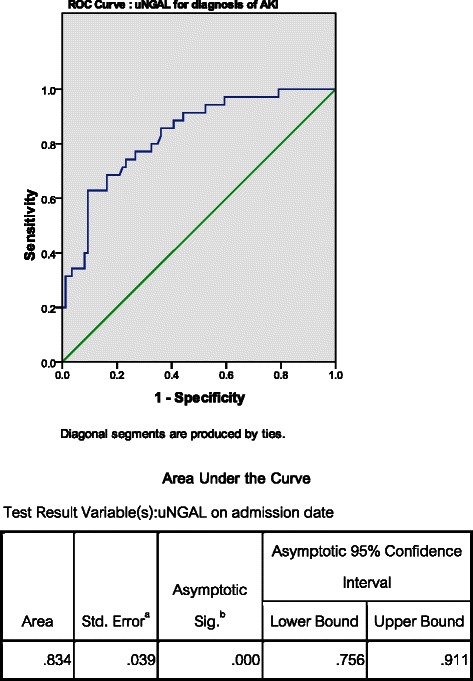
ROC curve present the uNGAL to diagnose AKI in hospitalized cirrhotic patients

For diagnosing AKI, the baseline uNGAL level upon admission was significantly higher in AKI than non-AKI subgroup (290.6 ± 356.3 vs. 54.4 ± 73.7 ng/mL; *P* = 0.0001). Previous study showed that uNGAL level might be increased due to UTI [25, 26]. Our subjects were further categorized according to the presence or absence of UTI as well as other bacterial infections. Patients with UTI (*n* = 11) had significantly higher uNGAL compared with non-UTI (256.9 ± 432.8 versus 109.3 ± 193.3 ng/mL, *P* = 0.04), whereas, patients with infections other than UTI (*n* = 53) did not have significant different of uNGAL level with non-infection group (148.4 ± 260.1 versus 102.8 ± 196.4 ng/mL, *P* = 0.28). We excluded UTI patients and performed the new analysis showing that uNGAL was still significantly higher in AKI versus non-AKI group (252 ± 295.8 versus 48.2 ± 64.3 ng/mL, *P* < 0.001) and similar as after excluding all bacterial infections, AKI group still had significantly higher uNGAL than non-AKI (130.5 ± 97.2 versus 57.6 ± 74.6 ng/mL, *P* = 0.007). This finding was also observed in previous study of cirrhotic patients (25). ROC curve showed that uNGAL level could be used to diagnose AKI in hospitalized cirrhotic patients with AKI-prone conditions with the AUC of 0.83 (95 % confidence interval [CI]: 0.76–0.91, *p* < 0.001) as shown in Fig. 2. The optimal cut-off value was 56 ng/mL providing 77.1 % sensitivity, 73.3 % specificity, 54 % positive predictive value (PPV), 88.7 % negative predictive value (NPV), respectively (Table 3). After excluding those with UTI, uNGAL still showed good diagnostic value for AKI (AUC = 0.84, 95 % CI 0.77–0.92, *P* < 0.001). In contrast, baseline serum creatinine performed poorly in diagnosing of AKI (AUC = 0.58, *P* = 0.4). In our study, there were no patients with hepatocellular carcinoma or other related malignancies which might influence the uNGAL level. In comparison of cirrhotic patients with cut-off uNGAL of 56 ng/mL, we found that those patients with uNGAL ≥56 ng/mL were significantly older, had higher serum creatinine, higher proportion of AKI and more number of death rate than those with uNGAL <56 ng/mL (Table 3).

**Table 3:** Patient characteristics at baseline categorized by the uNGAL cut off level of 56 ng/mL

Variables	Total	uNGAL <56 ng/mL	uNGAL ≥56 ng/mL	*P*
% or mean ± SD	(*n* = 121)	(*n* = 71; 58.7 %)	(*n* = 50; 41.3 %)	
Age (years)	57.3 ± 14.7	54.7 ± 13.8	61.0 ± 15.3	0.02*
% Male	75 (62 %)	46/71 (65 %)	29/50 (58 %)	0.45
Ascites	68 (56.2 %)	37/71 (52 %)	31/50 (62 %)	0.28
MELD score	14.9 ± 5.7	14.6 ± 6.2	15.4 ± 5.0	0.44
Serum creatinine	0.88 ± 0.29	0.82 ± 0.27	0.97 ± 0.32	0.006*
TB (mg/dl)	4.7 ± 5.1	4.3 ± 4.4	5.2 ± 6.2	0.34
DB (mg/dl)	3.4 ± 4.3	3.1 ± 3.4	3.9 ± 5.4	0.29
AST (U/L)	139.7 ± 250.3	148 ± 283	128 ± 196	0.66
ALT (U/L)	78.4 ± 174.5	74 ± 180	84 ± 168	0.76
ALP (U/L)	163.1 ± 108.1	155 ± 98	174 ± 121	0.35
INR	1.51 ± 0.77	1.4 ± 0.3	1.7 ± 1.1	0.12
Albumin (g/dl)	2.85 ± 0.86	2.8 ± 0.7	2.9 ± 1.0	0.75
Hemoglobin (g/dl)	10.7 ± 2.4	10.7 ± 2.5	10.7 ± 2.2	0.89
WBC count (10^3^/uL)	9.86 ± 6.2	9.9 ± 6.1	9.8 ± 6.3	0.94
Platelet (/10^3^uL)	128 ± 121	113 ± 66	146 ± 169	0.16
Presence of bacterial infection (%)	64/121 (52.8 %)	34/71 (48 %)	30/50 (60 %)	0.19
Length of stay (days)	9.9 ± 9.7	9.1 ± 9.6	11.0 ± 9.9	0.29
No AKI (%)	86/121 (71 %)	63/71 (89 %)	23/50 (46 %)	0.008*
Death (%)	17/121 (14 %)	5/71 (7 %)	12/50 (24 %)	<0.0001*

*P < 0.05

For AKI-subtype differentiation, a total of 35 hospitalized cirrhotic patients who developed AKI, 24 (68.6 %) were diagnosed with PRA, 2 (5.7 %) with HRS, and 9 (25.7 %) with ATN, respectively. The mean uNGAL level was higher in the patients with ATN than in those with HRS and prerenal azotemia, respectively (639.7 ± 532.2 vs. 241.0 ± 41.0 vs. 163.8 ± 156.2 ng/mL, *P* = 0.001). The median uNGAL levels of the ATN and non-ATN groups were 559 and 156 ng/mL, respectively (*P* = 0.005). At the optimal uNGAL cut-off value of 136.8 ng/mL, a sensitivity and specificity of 88.9 % and 80.4 %, respectively, was achieved for the diagnosis of ATN, with an AUC-ROC of 0.91 (95 % CI: 0.83–0.98, *P* < 0.05) (Table 4).

**Table 4:** The discriminative value of uNGAL

uNGAL (ng/mL)	AUC	95 % CI	*P*	Cut-off ng/mL	Sensitivity (%)	Specificity (%)	LR+	LR-
AKI vs non-AKI	0.83	0.76–0.91	<0.001	56	77.1	73.3	2.85	0.32
ATN vs non-ATN	0.91	0.83–0.98	<0.001	136.8	88.9	80.4	4.54	0.14
30-day mortality vs survival group	0.75	0.66–0.85	0.001	72	70.6	69.2	2.29	0.42

### uNGAL and the prognostic value

Seventeen patients (14 %) died during the 30-day follow-up period. The 30-day mortality rate was significantly higher in AKI group than those without AKI (31.4 % vs. 7.0 %, *P* < 0.05). The major causes of death were multiple organ failures and sepsis. The mean uNGAL level was significantly higher in the mortality group compared with the survivor group (217.7 vs. 107.2 ng/mL, *P* = 0.03). The AUC (95 % CI) for uNGAL for predicting 30-day mortality was 0.75 (0.66–0.85, *P* = 0.001), with a best cut-off value of 72 ng/mL providing 70.6 % sensitivity and 69.2 % specificity (Table 5).

**Table 5:** Univariate and multivariate logistic regression analysis demonstrating the relationship of 30-day mortality with other variables

	Univariate		Multivariate	
	OR (95 % CI)	*P*	OR (95 % CI)	*P*
Age	1.05 (1.01–1.1)	0.01	1.05 (1.00–1.1)	0.05*
Bacterial infection	0.12 (0.14–1.27)	0.42		
Vasopressor	0.20 (0.06–0.64)	0.007	0.43 (0.10–1.85)	0.26
Blood transfusion	0.76 (0.27–2.2)	0.60		
Hypovolemia	0.86 (0.31–2.4)	0.77		
Blood culture	0.28 (0.1–0.82)	0.02	0.52 (0.15–1.83)	0.31
Length of stay	1.04 (0.99–1.08)	0.08		
Ascites	0.66 (0.23–1.92)	0.45		
AKI**	0.16 (0.06–0.49)	0.001	0.28 (0.08–1.02)	0.05*
Serum creatinine	2.03 (0.38–10.79)	0.41		
uNGAL > 72 ng/mL	0.23 (0.08–0.68)	0.008	0.63 (0.17–2.31)	0.49
Child-Pugh class C	1.08 (0.37–3.15)	0.90		
MELD** score	1.05 (0.97–1.15)	0.24		

*P ≤ 0.05, MELD**:Model for End stage Liver Disease, AKI**:Acute kidney injury

We used univariate logistic regression analysis to evaluate the association of age, clinical variables, laboratory values, and biomarkers with 30-day mortality. Age, the use of vasopressor, positive blood culture, AKI and uNGAL >72 ng/mL were significantly associated with 30-day mortality, whereas, bacterial infection, blood transfusion, hypovolemia, length of hospital stay, ascites, serum creatinine, Child-Pugh class C, and MELD score were not. These variables with *P* < 0.2 in univariate logistic regression model including age, presence of bacterial infection, MELD score, hemoglobin level, length of hospital stay, presence of AKI, uNGAL on admission, vasopressor use and renal replacement therapy were added into multivariate logistic regression analysis. We found that uNGAL was not the independent factor to predict 30-day mortality adjusting to other factors (Table 4).

## Discussion

This is the first prospective study investigate the cut-off level of uNGAL for diagnosing new-onset AKI in hospitalized cirrhotic patients with AKI-prone conditions. The data suggest that a single measurement within 24 h after admission is able to diagnose AKI as we measured twice 24-hour apart and showed good correlation. This finding confirms the previous results that were established in other patient settings with AKI that could be conveniently applied in cirrhotic patients [12, 27].

Our study design is different from former studies as we investigated cirrhotic patients with normal baseline serum creatinine who developed AKI during admission. Those patients associated with AKI-prone conditions that frequently finds in clinical practice. For diagnosing AKI, our study observed the markedly higher uNGAL in AKI patients compared with non-AKI, which was consistent with previous study in cirrhotic patients [17]. As UTI might affect higher uNGAL [25, 26], our study also confirmed this finding after excluded both bacterial infection and UTI subgroups and re-analysis the data. uNGAL is a good biomarker for early diagnosis with high AUC-ROC (0.83) with cut-off value of 56 ng/mL providing a good sensitivity and specificity. This level might be used for the early detection of AKI in hospitalized AKI-prone cirrhotic patients, for example, those with bacterial infection, acute decompensated cirrhosis or GI bleeding, which occurred frequently during admission. Previous studies validated cut-off level of uNGAL in other settings including hematopoietic stem cell transplantation, critically ill and after coronary angiography, which were the high risks for AKI. Those studies’ cutoff provided good diagnostic efficacy and were superior to other kidney biomarkers such as urinary kidney injury molecule-1, liver-type fatty acid-binding protein [28–30]. Our study extended these observations to specific group of cirrhotic patients. Former study showed that the severity of renal injury in hospitalized cirrhotic patients related with poor clinical outcome and early treatment could provide better result. Therefore, it is crucial to early detect AKI with effective marker in order to provide early intervention [2, 6].

Cirrhotic patients might be complicated with HRS, which is difficult to distinguish with ATN clinically [31]. HRS is characterized by marked renal vasoconstriction with a consequence of reduced glomerular function with preserved tubular function [32]. Therefore, uNGAL level should not be high as this marker expresses in renal tubule [33]. Our study confirmed that uNGAL was significantly higher in ATN compared with non-ATN and its level could differentiate ATN from other subtypes with AUC of 0.91. This finding was consistent with other previous studies [14, 16]. It is crucial to differentiate between subtypes of AKI because of the difference in management. HRS is treated with vasoconstrictor, albumin infusion and TIPS placement, whereas; ATN should be treated with renal replacement therapy.

In term of the early predicting outcome of this marker, there were few recent studies investigated in cirrhotic patients showing that uNGAL was the independent factor to predict mortality in different settings [15, 34], whereas the other study was not [19]. Our study found that uNGAL was significantly higher in 30-day mortality group compared with survival group and performed well in predicting the outcome with AUC value of 0.75. This AUC was comparable to previous cohort [19]. However, after adjusting to other factors in multivariate logistic regression model, uNGAL was not an independent factor associated with the 30-day mortality. This finding differed from previous studies in cirrhotic patients and might be contributed to the adjusting factors that we included in the multivariate model and the more focus for only liver-related mortality. The severity of Child-Pugh grading did not significantly predict death in our study; this finding may be explained that the major causes of death in our cirrhotic patients were not liver failure. Previous study by Moreau R and colleagues showed that cirrhotic patients with acute on chronic liver failure (ACLF) had 28-day mortality rate of 33.9 %[35] which was similar to our study, reporting the 30-day mortality rate of cirrhotic patients with AKI of 31.4 %. In addition, the higher numbers of major organs failures was a good predictor of higher mortality rate in cirrhotic patients with ACLF [35]. Recent study showed that the precipitating factors from hepatic or extrahepatic causes may influence on the difference of ACLF clinical course and prognosis [36].

There were some limitations in our study. First, AKI was diagnosed by AKIN criteria that based on serum creatinine, which is underestimated in cirrhotic patients and might be inaccurate. However, serum creatinine is the marker that is the most widely used in clinical practice. Second, our study contained a relatively small sample size of AKI, especially the HRS subgroup, which might limit the interpretation of the differentiation between subtypes of AKI. Third, our study did not measure other renal biomarkers for comparison of the efficacy with uNGAL. Finally, our study lacks of the validation cohort which needs to test in the future.

## Conclusion

Our prospective study indicates that uNGAL is a valid marker for the early detection of AKI in cirrhotic patients with AKI-prone conditions. With a cut-off value of 56 ng/mL, it provides an AUC of 0.83, with a sensitivity and specificity of 77.1 and 73.3 %, respectively. In addition, its level can differentiate between ATN and non-ATN subtype. However, this marker did not independently predict 30-day liver-related mortality.

## Abbreviations

AKI: Acute kidney injury
AKIN: Acute kidney injury network
ATN: Acute tubular necrosis
AUC: Area under the curve
GI: Gastrointestinal
HRS: Hepatorenal syndrome
MELD: Model for end-stage liver disease
PRA: Prerenal azotemia
ROC: Receiver operating characteristic
SBP: Spontaneous bacterial peritonitis
SD: Standard deviation
uNGAL: Urine neutrophil gelatinase-associated lipocalin
UTI: Urinary tract infection
vs: Versus
